# M-Bonacci Zone Plates for Ultrasound Focusing

**DOI:** 10.3390/s19194313

**Published:** 2019-10-05

**Authors:** Sergio Pérez-López, José Miguel Fuster, Pilar Candelas

**Affiliations:** Centro de Tecnologías Físicas, Universitat Politècnica de València, 46022 València, Spain; serpelo1@teleco.upv.es (S.P.-L.); pcandelas@fis.upv.es (P.C.)

**Keywords:** M-bonacci zone plate, Fresnel zone plate, acoustic focusing, acoustic lens

## Abstract

In this work, we present a thorough analysis on M-bonacci zone plates for ultrasound focusing applications. These planar lenses are capable of providing bifocal focusing profiles with equal intensity in both foci and become very appealing for a wide range of scenarios including medical and industrial applications. We show that in high-wavelength domains, such as acoustics or microwaves, the separation between both foci can be finely adjusted at the expense of slightly increasing the distortion of the focusing profile, and we introduce a design parameter to deal with this issue and simplify the design process of these lenses. Experimental measurements are in good agreement with numerical simulations and demonstrate the potential of M-bonacci lenses in ultrasound focusing applications.

## 1. Introduction

Acoustic lenses are devices capable of focusing incident sound waves into specific focal areas. Due to their wide range of applications, including both industrial and medical, acoustic lenses are a hot topic among the scientific community. Nowadays, approaches to focus acoustic waves are mainly based on metasurfaces, which can be implemented with subwavelength slits [1], coiling-up space structures [2,3,4], or Helmholtz resonators [5]. Holographic structures have also been proposed as a flexible solution for synthesis of focusing profiles [6]. However, these kind of devices are usually difficult to design because they require a complete 3D design of each of their unit cells. Other focusing alternatives are based on either spherical [7] or cylindrical [8] containers filled with liquids, where the acoustic properties of the inner liquid determines the lens focusing profile. Although these type of lenses can be very interesting in certain applications, and they have shown focal tunability [7], they can only focus in very close range applications, which limits their potential. In this sense, a simpler device capable of focusing acoustic waves with higher flexibility is the Fresnel Zone Plate (FZP), which can be implemented either alternating blocking or absorbing regions with transparent regions [9,10], or alternating transparent with phase-reversal regions [11].

FZPs have been widely used in many areas of physics due to their advantageous planar fabrication compared to conventional curved lenses. A FZP focuses waves through constructive interference of the diffracted field in its apertures. These devices can be found in all sorts of applications, such as optical trapping [12], planar antenna design [13], or ultrasound focusing applications [9,10,14]. In optics, the distance between the transducer and the lens is usually large enough to consider plane wave incidence. However, in many ultrasound and microwave applications, a directional emitter is placed at a distance where the plane wave approximation is not valid. Therefore, to properly obtain the focusing profile of an acoustic lens, the influence of the transducer on the lens energy distribution has to be considered [10,15].

In the past years, several lenses with interesting focusing properties have been introduced in the optical domain, such as Fibonacci ZPs [16,17], Cantor ZPs [18,19,20], and Thue-Morse ZPs [21,22]. All these novel ZPs are variations over conventional FZPs, where transparent and opaque/phase-reversal Fresnel regions are distributed according to a specific binary sequence [23]. M-bonacci Zone Plates (MbZPs) [17] are based on M-bonacci sequences, a generalization of the well-known Fibonacci sequence. They produce bifocal focusing profiles with equal intensity focus at distances related to the M-bonacci sequence ratio. The application of ultrasound focusing techniques is a topic of great interest in both photoacoustic imaging [24,25] and therapeutic applications [26,27,28,29]. In the latter, dual focusing profiles can be used to either treat two isolated regions simultaneously or target extensive areas with higher efficiency [30,31].

Although M-bonacci sequences have been previously proposed for ultrasound focusing applications [23], in this work, we experimentally demonstrate the feasibility of MbZPs in the ultrasound domain for the first time. Moreover, we analyze the distortion introduced in the focusing profile by these type of lenses in high-wavelength domains, such as acoustics or microwaves, when the Fresnel approximation is not fulfilled, introducing a new parameter, which becomes very helpful at the design stage and allows a fine adjustment of the separation between both foci at the expense of slightly distorting the MbZP focusing profile.

## 2. M-Bonacci Zone Plates

### 2.1. Design and Focusing Properties

MbZPs are formed by aperiodic binary sequences based on M-bonacci series. To build a M-bonacci serie of order *m* and stage *j*, two initiators of the sequence are defined as in the Fibonacci case, $F_{m,1}=0$ and $F_{m,2}=1$. The next numbers of the series are calculated as the sum of the *m*-previous numbers:

(1) $$F_{m,j+1}=\left\{\begin{array}{cc} \sum_{i=1}^{j}F_{m,i} & ,j\leq m\\ \sum_{i=j-m+1}^{j}F_{m,i} & ,j>m \end{array}\right..$$

Table 1 shows the first ten numbers of the M-bonacci series calculated for m=2 (Fibonacci case), m=3 and m=4.

One interesting parameter that can be derived from the M-bonacci series is the sequence ratio, $\varphi_{m}$, defined as the limit of the ratio between two consecutive numbers:

(2) $$\varphi_{m}=\lim_{j\to +\infty }\left(\frac{F_{m,j+1}}{F_{m,j}}\right).$$

When m=2, the ratio corresponds to the golden mean $\varphi_{2}=1.618$, whereas for m=3 and m=4, the sequence ratios are $\varphi_{3}=1.839$ and $\varphi_{4}=1.928$, respectively.

Analogously to the M-bonacci series, a binary M-bonacci sequence can be formed using the initiators $f_{m,1}=0$ and $f_{m,2}=1$. The next instance of the M-bonacci sequence is obtained as the concatenation of the *m*-previous M-bonacci instances, using the following equation,

(3) $$f_{m,j+1}=\left\{\begin{array}{cc} \left\{f_{m,j}\;\&\;f_{m,j-1}\;\&\;\ldots \;\&\;f_{m,1}\right\} & ,j\leq m\\ \left\{f_{m,j}\;\&\;f_{m,j-1}\;\&\;\ldots \;\&\;f_{m,j-m+1}\right\} & ,j>m \end{array}\right.$$

where “&” represents the concatenation operator.

Table 2 shows the first six iterations of M-bonacci binary sequences for m=2, m=3, and m=4. In each sequence, there are $F_{m,j+1}$ binary elements, being $F_{m,j}$ type-1 and $F_{m,j+1}-F_{m,j}$ type-0. The notation regarding type-1 and type-0 elements is used to indicate whether the binary element is either a 1 or a 0, and thus determines how the corresponding Fresnel region is implemented. In Soret ZPs, type-1 elements are implemented as opaque regions, whereas type-0 elements correspond to transparent regions. As an example, the binary sequence $f_{2,6}=\left\{10110101\right\}$, corresponding to m=2 and j=6, indicates that the first, third, fourth, sixth, and eight Fresnel regions of the lens are opaque regions, whereas the rest (second, fifth, and seventh regions) are transparent. The number of opaque (type-1), transparent (type-0), and total Fresnel regions can also be verified using the numbers of the M-bonacci series shown in Table 1, resulting, for this particular example, in a total of $F_{m,j+1}=F_{2,7}=8$ regions, with $F_{2,6}=5$ type-1 opaque regions and $F_{2,6}-F_{2,5}=3$ type-0 transparent regions. The limit of the ratio between the number of type-1 and type-0 elements is given by

(4) $$\tau_{m}=\lim_{j\to +\infty }\left(\frac{F_{m,j}}{F_{m,j+1}-F_{m,j}}\right)=\frac{1}{\varphi_{m}-1}.$$

As stated before, M-bonacci ZPs are obtained by applying a binary sequence to a conventional FZP. Once the M-bonacii binary sequence has been calculated, the MbZP is obtained assigning type-1 and type-0 elements to the corresponding Fresnel regions of its associated FZP. When plane wave incidence is considered, the radii of the different Fresnel regions are given by

(5) $$r_{n}=\sqrt{n\lambda z_{0}+{\left(\frac{n\lambda }{2}\right)}^{2}},$$

where λ represents the wavelength, $z_{0}$ represents the focal distance, and n=1,2,…,N, with *N* being the total number of Fresnel regions.

**Figure 1:**
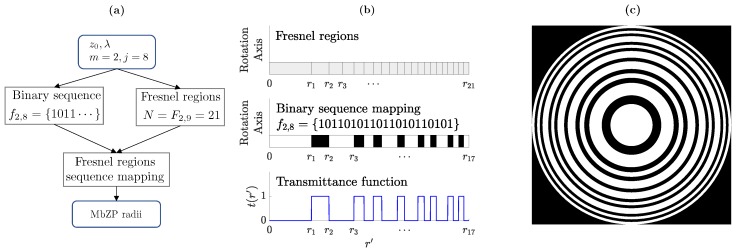
Lens design procedure with m=2 and j=8: (**a**) design steps; (**b**) Fresnel regions (top), classification of M-bonacci Zone Plate (MbZP) regions after the binary sequence mapping (middle) and final transmittance function of the lens (bottom); and (**c**) resulting MbZP layout.

Figure 1 shows a MbZP design example with m=2 and j=8. The flow diagram of the lens design procedure is depicted in Figure 1a. Figure 1b shows the distribution of the Fresnel regions for the current example and their mapping using the M-bonacci sequence distribution. Finally, Figure 1c depicts the final MbZP layout resulting from the lens design.

Once the M-bonacci ZP radii are obtained, the focusing profile of the lens can be calculated by numerically computing the Rayleigh–Sommerfeld diffraction integral,

(6) $$I\left(z\right)=\frac{4\pi^{2}}{\lambda^{2}}{\left|\int_{0}^{a}p_{i}\left(r^{\prime }\right)t\left(r^{\prime }\right)\frac{e^{-jkr}}{r}\cos\left(n,r\right)r^{\prime }dr^{\prime }\right|}^{2},$$

where *a* is the maximum radius of the lens, $r^{\prime }$ is the radial coordinate of the lens, k=2π/λ is the wavenumber, $p_{i}\left(r^{\prime }\right)$ is the incident pressure distribution, $t(r^{\prime })$ is the ZP transmittance function, $r=\sqrt{{\left(r^{\prime }\right)}^{2}+z^{2}}$, *z* is the axial coordinate and cos(n,r)=z/r, with *n* being the normal direction to the lens surface. For a Soret ZP, the transmittance function, also known as pupil function, is 0 at the pressure blocking regions and 1 at the transparent regions.

In Equation (6), the distance *r* can be expressed as

(7) $$r=z\sqrt{1+{\left(\frac{r^{\prime }}{z}\right)}^{2}}.$$

Using the Taylor expansion $\sqrt{1+x}≅1+x/2-x^{2}/8$, *r* can be approximated as

(8) $$r≅z+\frac{{\left(r^{\prime }\right)}^{2}}{2z}-\frac{{\left(r^{\prime }\right)}^{4}}{8z^{3}}.$$

The Fresnel approximation assumes that the third term of the Taylor expansion of *r* does not affect the phase of the exponential term, which means that its contribution to the exponent has to be much lower than 2π, that is,

(9) $$k\frac{{\left(r^{\prime }\right)}^{4}}{8z^{3}}<<2\pi .$$

The worst case is obtained when $r^{\prime }$ reaches its maximum value at $r^{\prime }=a$, which means that the Fresnel approximation stands for axial coordinates greater than

(10) $$z^{3}>>\frac{a^{4}}{8\lambda }.$$

If the above condition is met, $r≅z+\frac{{\left(r^{\prime }\right)}^{2}}{2z}$, and Equation (6) can be approximated as

(11) $$I\left(z\right)≅\frac{4\pi^{2}}{\lambda^{2}}{\left|\int_{0}^{a}p_{i}\left(r^{\prime }\right)t\left(r^{\prime }\right)\frac{e^{-jkz}e^{-jk\frac{{\left(r^{\prime }\right)}^{2}}{2z}}}{z+\frac{{\left(r^{\prime }\right)}^{2}}{2z}}\cos\left(n,r\right)r^{\prime }dr^{\prime }\right|}^{2}.$$

Further approximations can be done, as the paraxial approximation is valid ($z>>r^{\prime }$) and therefore cos(n,r)≅1 and the denominator at the integral becomes $z+\frac{{\left(r^{\prime }\right)}^{2}}{2z}≅z$, which results in

(12) $$I\left(z\right)≅\frac{4\pi^{2}}{\lambda^{2}z^{2}}{\left|\int_{0}^{a}p_{i}\left(r^{\prime }\right)t\left(r^{\prime }\right)e^{-jk\frac{{\left(r^{\prime }\right)}^{2}}{2z}}r^{\prime }dr^{\prime }\right|}^{2}$$

It is convenient to implement a variable change in both axial and radial directions. Thus, a variable $u=\frac{a^{2}}{2\lambda z}$ is defined as the normalized axial coordinate, whereas a variable $\xi ={\left(\frac{r^{\prime }}{a}\right)}^{2}$ is defined as the normalized radial coordinate. Keeping in mind that $dr^{\prime }=\frac{a}{2\sqrt{\xi }}d\xi$, Equation (12) becomes

(13) $$I\left(u\right)≅4\pi^{2}u^{2}{\left|\int_{0}^{1}p_{i}\left(\xi \right)t\left(\xi \right)e^{-j2\pi \xi u}d\xi \right|}^{2},$$

which means that if the Fresnel approximation condition is fulfilled (Equation (10) is verified), the focusing profile of the lens can be obtained as a Fourier transform of the pressure distribution at the ZP aperture. When the Fresnel approximation is not applicable and Equation (13) cannot be used, the focusing profile of the MbZP is computed using Equation (6) and presents a certain distortion compared to the ideal case.

For a standard FZP, the focal distance in the normalized axial coordinate can be calculated using Equation (5) as

(14) $$u_{0}=\frac{a^{2}}{2\lambda z_{0}}=\frac{N\lambda z_{0}+{\left(\frac{N\lambda }{2}\right)}^{2}}{2\lambda z_{0}}=\frac{N}{2}+\frac{N^{2}\lambda }{8z_{0}}=\frac{N}{2}\left(1+\varepsilon \right),$$

being $a=r_{N}$ the maximum radius of the lens and $\varepsilon =\frac{N\lambda }{4z_{0}}$.

For low wavelength domains, such as optics, ε<<1, and therefore Equation (14) can be approximated as

(15) $$u_{0}≅\frac{N}{2}.$$

Interestingly, as reported by Monsoriu et al. [16,17], the focusing profile of MbZPs shows two symmetric foci respect to the normalized focal distance of its associated FZP ($u_{0}=N/2=F_{m,j+1}/2$). The first focus is located at $u_{1}≅F_{m,j}$, which corresponds to the number of type-1 elements in the binary sequence, whereas the second focus is located at $u_{2}≅F_{m,j+1}-F_{m,j}$, which corresponds to the number of type-0 elements in the sequence. Therefore, the ratio between the two foci tends to $u_{1}/u_{2}≅\tau_{m}$, as stated in Equation (4). The focal positions in the denormalized axial coordinate can be calculated as

(16) $$z_{1}=\frac{F_{m,j+1}}{F_{m,j}}\cdot \frac{z_{0}}{2}≅\varphi_{m}\frac{z_{0}}{2},$$

and

(17) $$z_{2}=\frac{F_{m,j+1}}{F_{m,j+1}-F_{m,j}}\cdot \frac{z_{0}}{2}≅\varphi_{m}\tau_{m}\frac{z_{0}}{2},$$

$z_{0}$ being the focal distance of the associated FZP.

**Figure 2:**
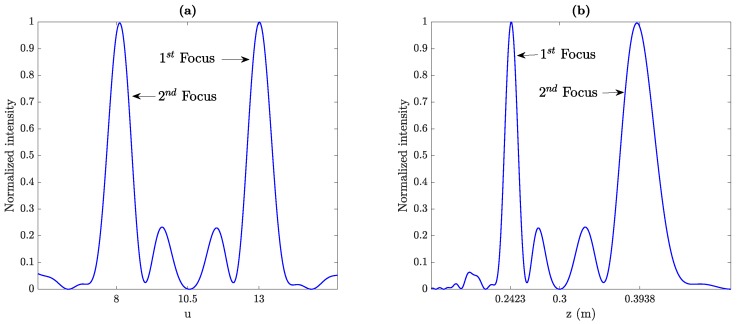
Focusing profile of a lens with m=2, j=8, $z_{0}=0.3$ m, and λ=0.3 mm as a function of (**a**) the normalized axial coordinate and (**b**) the denormalized axial coordinate.

Figure 2a,b shows the focusing profile of a MbZP lens against the normalized (*u*) and denormalized (*z*) axial coordinate, respectively. As it can be observed from Figure 2a, when using the normalized axial coordinate, the MbZP is completely symmetrical as it was previously shown in optics [16]. The normalized focal distances are $u_{1}≅F_{2,8}=13$ and $u_{2}≅F_{2,9}-F_{2,8}=8$, which agrees with their theoretical values. On the other hand, when the axial coordinate is denormalized and the real focusing profile is depicted (Figure 2b), the foci are not symmetrical anymore, due to the coordinate transformation that relates *u* with *z*. The main effect over the focusing profile is that the focus further from the lens becomes wider than the focus closer to the lens.

**Figure 3:**
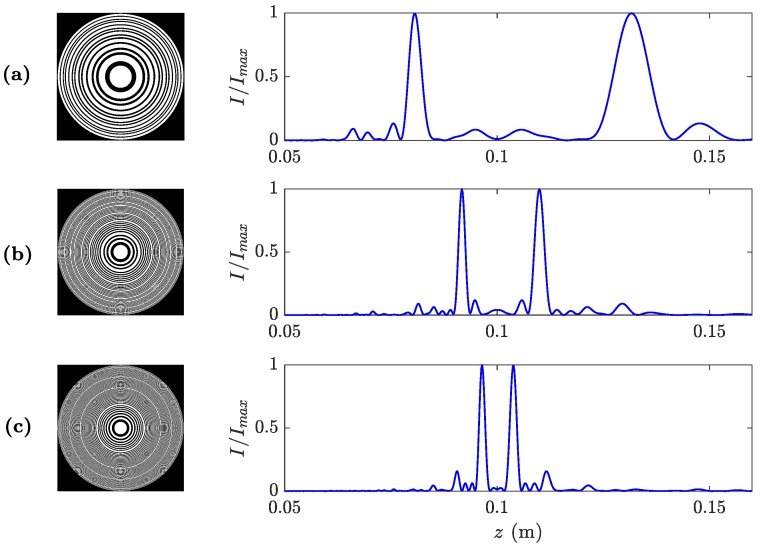
MbZP layouts (left) and their normalized focusing profiles (right): (**a**) m=2, (**b**) m=3, and (**c**) m=4. For all MbZPs, j=9 and $z_{0}=0.1$ m.

Figure 3 depicts the computed focusing profiles along the axial coordinate for three different MbZPs. Figure 3a corresponds to m=2, whereas Figure 3b,c correspond to m=3 and m=4, respectively. As it can be observed from Figure 3a, $z_{1}≅8.1$ cm and $z_{2}≅13.1$ cm, which agree with their theoretical focal distances obtained from Equations (16) and (17). Figure 3b shows two foci at $z_{1}≅9.2$ cm and $z_{2}≅11.0$ cm, whereas Figure 3c shows two foci at $z_{1}≅9.6$ cm and $z_{2}≅10.4$ cm, which also agrees with the theory.

### 2.2. γ-Parameter

In high-wavelength domains, such as ultrasounds, the Fresnel approximation can not always be assumed in typical focusing applications. Therefore, it is useful to define a γ parameter as the ratio between the associated FZP focal distance, $z_{0}$, and the Fresnel approximation distance, $z_{f}$, given by

(18) $$\gamma =\frac{z_{0}}{z_{f}},$$

where $z_{f}=\sqrt[{3}]{\frac{a^{4}}{8\lambda }}$.

**Figure 4:**
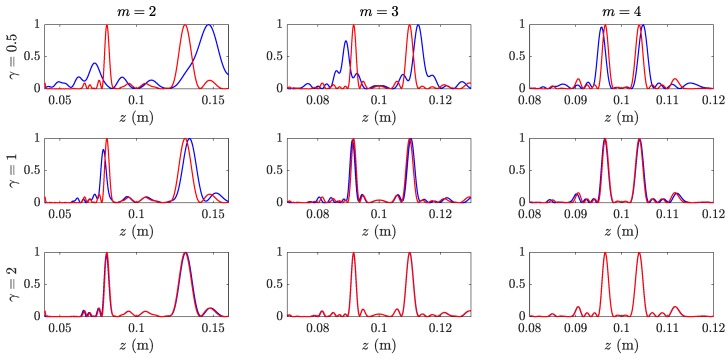
Focusing profiles (blue lines) against axial distance for different values of γ (rows) and *m* (columns), compared to their ideal counterparts (γ>>1) in red.

Thus, the Fresnel approximation is valid when γ>>1. In high-wavelength domains, fulfilling this condition requires very long focal distances, which is not the usual case in most near-field focusing applications. However, simulations show that MbZPs present low distortion for γ>2 values, which is a more reasonable condition to achieve. In fact, MbZPs can be designed in the 1<γ<2 range with low distortion in the focusing profile. Alternatively, if γ<1, MbZPs present a highly distorted focusing profile. This distortion results in focal displacement and focal intensity reduction compared to the γ>>1 case. Figure 4 depicts the numerically computed axial intensities using Equation (6) for MbZPs (blue lines) with different γ and *m* values, compared to the case when γ>>1, and the Fresnel approximation is valid (red lines). As it can be observed from Figure 4, when γ=0.5 (first row) the distortion is very severe, and the focal distances in all three cases do not correspond to the theoretical values. When γ=1, the distortion is significantly reduced in comparison with the γ=0.5 case, and the focusing profiles are closer to the theoretical value. Finally, when γ=2 (third row) minimum distortion is observed.

Thus, the γ parameter should be considered in the design process of MbZPs. Combining Equation (5) with the Fresnel approximation distance condition results in

(19) $$z_{f}^{3}=\frac{{\left(F_{m,j+1}z_{0}\right)}^{2}}{8}\lambda {\left(1+\varepsilon \right)}^{2}.$$

Using Equations (18) and (19), and substituting $\varepsilon =\frac{N\lambda }{4z_{0}}$, yields to the following third-order equation,

(20) $$\frac{1}{16}\gamma^{3}F_{m,j+1}^{4}\psi^{3}+\frac{1}{2}\gamma^{3}F_{m,j+1}^{3}\psi^{2}+\gamma^{3}F_{m,j+1}^{2}\psi -8=0,$$

where $\psi =\frac{\lambda }{z_{0}}$ is an additional design variable.

Therefore, once ψ has been numerically calculated, solving Equation (20) to achieve a specific γ parameter value, it is possible to obtain the focal distance for a given wavelength or the required wavelength for a specific focal distance.

**Figure 5:**
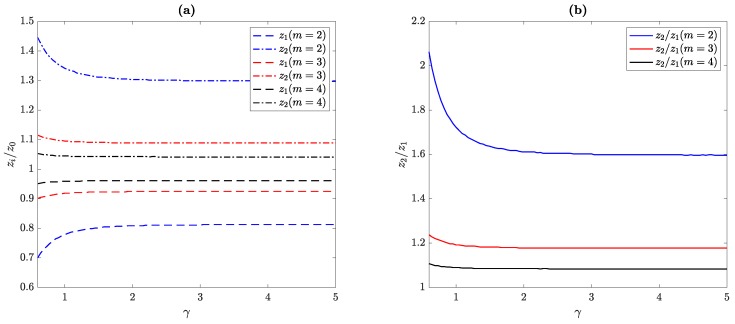
(**a**) Normalized focal distances and (**b**) focal distance ratio as a function of the γ parameter.

Figure 5a depicts the normalized focal distances for the first and second focus against the γ parameter for three different cases: m=2 (red lines), m=3 (blue lines), and m=4 (black lines). Figure 5b shows the focal distance ratio, defined as $z_{2}/z_{1}$, for the same three cases. The results have been obtained using Equation (6). All MbZPs have been designed for a fixed $z_{0}$, whereas the wavelength has been adjusted using Equation (20) in each simulation to provide the desired γ factor. As it can be observed from Figure 5, for γ>2, the focal distances of both foci tend to their theoretical positions (γ→+∞ case), whereas γ<2 results in both foci being shifted away in opposite directions. This phenomenon can be easily understood by observing the focal distance ratio, which theoretically tends to $z_{2}/z_{1}≅\tau_{m}$, according to Equations (16) and (17). Thus, as the γ parameter augments, $z_{2}/z_{1}$ tends to 1.60, 1.18, and 1.08 for m=2, m=3, and m=4, respectively, which are very close to the theoretical values given by $\tau_{2}=1.618$, $\tau_{3}=1.192$, and $\tau_{4}=1.078$. Moreover, when γ<2, the displacement on the focal distances is more noticeable in the m=2 case, which agrees with the results depicted in Figure 4.

**Figure 6:**
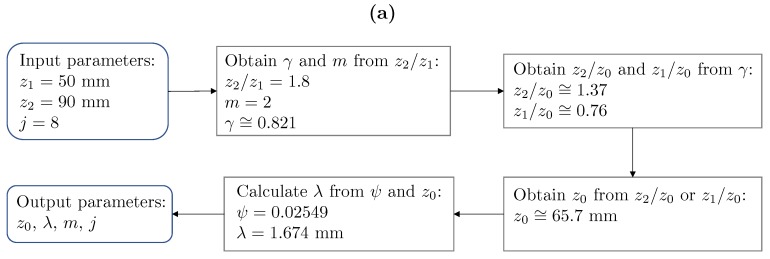

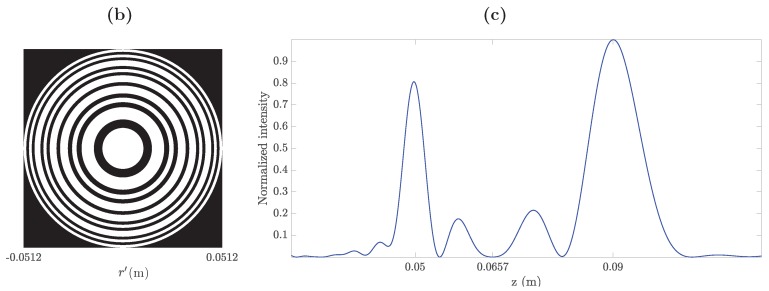
Lens design example with $z_{1}=50$ mm and $z_{2}=90$ mm: (**a**) Design procedure flow diagram, (**b**) resulting MbZP lens, and (**c**) numerically computed focusing profile.

Figure 6 illustrates a MbZP design example using the tools developed in this work. Figure 6a shows the concept diagram with the different steps required to accurately design the MbZP. Initially, the input parameters are the desired location of both foci, $z_{1}$ and $z_{2}$, and the resolution level of the lens, which is related with the MbZP size and, thus, with the *j* parameter. In this particular example, these three parameters have been selected as $z_{1}=50$ mm, $z_{2}=90$ mm, and j=8. In the next design step, both the order of the M-bonacci sequence *m* and the required γ parameter are found by computing the focal distance ratio, $z_{2}/z_{1}$, and then using Figure 5b to retrieve them. With *m* and γ, the normalized focal distances, $z_{1}/z_{0}$ and $z_{2}/z_{0}$, can be obtained using Figure 5a, and therefore $z_{0}$ is found. Using Equation (20), the ψ parameter can be found, and λ is finally obtained as the product $z_{0}\psi$ to determine the design frequency for the MbZP lens.

Once the design parameters *m*, *j*, λ, and $z_{0}$ have been determined, the MbZP radii can be calculated, as it was previously shown in Figure 1a, and the MbZP lens can be built. Figure 6b shows the resulting MbZP layout for this particular design example with m=2, j=8, λ=1.67 mm, and $z_{0}=65.7$ mm, and Figure 6c depicts the MbZP focusing profile with the first and second foci located at positions $z_{1}=50$ mm and $z_{2}=90$ mm as required, demonstrating the feasibility and utility of this design method.

## 3. Experimental Results and Discussion

**Figure 7:**
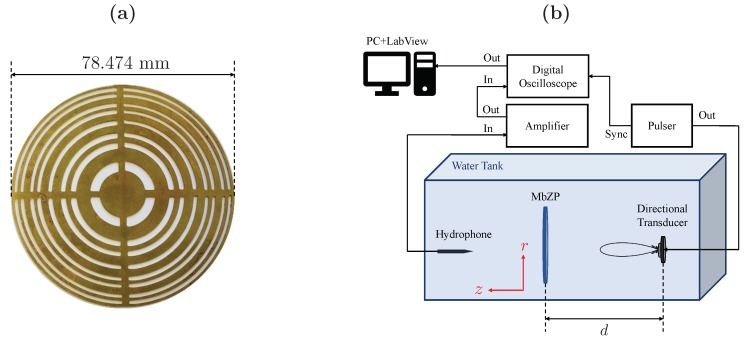
(**a**) Manufactured MbZP and (**b**) scheme of the experimental set-up.

Experimental measurements have been carried out in order to validate the theoretical analysis. The experimental set-up consists of an underwater 3D automated positioning system with a spatial resolution of 1×1×1 mm${}^{3}$. An Imasonic piston transducer with 12.7 mm of active diameter and a central working frequency of 1 MHz is used as emitter. A needle hydrophone form Precision Acoustics Ltd. with 1.5 mm of diameter and a −4 dB bandwidth ranging from 200 kHz to 25 MHz is used as receiver. The transmitted signal is generated using a Panametrics 5077PR pulser and sampled using a digital oscilloscope from Pico Technology with a resolution of 12-bit. Figure 7b depicts a scheme of the experimental set-up.

As plane wave incidence cannot be assumed due to the reduced physical dimensions of the water tank, the Fresnel radii were calculated using the spherical wave incidence equation, given by

(21) $$d+z_{0}+\frac{n\lambda }{2}=\sqrt{d^{2}+r_{n}^{2}}+\sqrt{z_{0}^{2}+r_{n}^{2}},$$

where d=350 mm is the transducer separation from the lens. To obtain the γ parameter under spherical wave incidence, an iterative process is used. In this process, the ψ value for plane wave incidence is used to obtain an initial value of λ. Subsequently, the wavelength is iteratively increased until the desired γ value is achieved.

**Figure 8:**
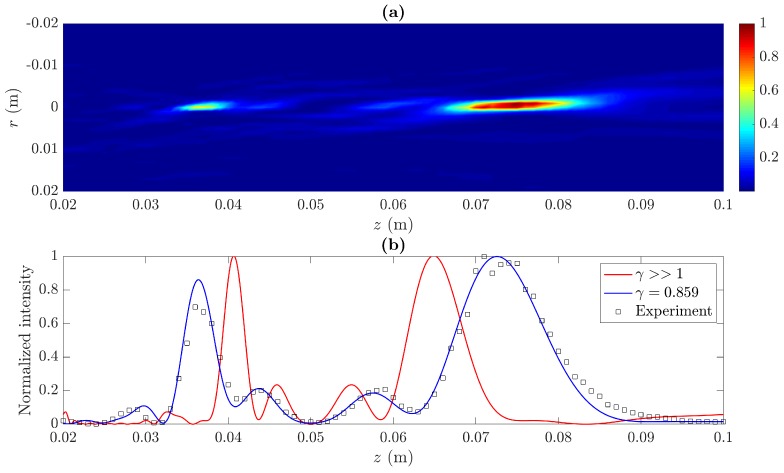
Experimental results: (**a**) measured intensity map, (**b**) measured (black squares) and simulated (blue line) focusing profiles for the MbZP built lens compared to the γ>>1 case (red line).

A Soret MbZP, made of brass and designed with m=2, j=8, $z_{0}=5$ cm, and λ=1.5 mm, has been manufactured (Figure 7a). In this lens, the Fresnel approximation distance is $z_{f}=5.824$ cm and, therefore, γ=0.859, meaning that the focusing profile of the lens is going to be significantly distorted. Figure 8a depicts the measured acoustic intensity map, whereas Figure 8b shows the measured focusing profile (squares) along the axial distance compared to the numerical simulation (blue line) and the γ>>1 case (red line).

As it can be observed, the manufactured lens with γ=0.859 provides focal distances of $z_{1}=3.64$ cm and $z_{2}=7.26$ cm, which differ from those corresponding to the γ>>1 case ($z_{1}=4.06$ cm and $z_{2}=6.49$ cm). Therefore, the experimental results demonstrate the focal shift predicted in the theoretical analysis and shown in Figure 4 and Figure 5. However, there is also a noticeable distortion in the focusing profile, and both foci are not even anymore. Thus, there is a trade off between the focal shifting range and the maximum tolerable distortion in the MbZP focusing profile.

**Figure 9:**
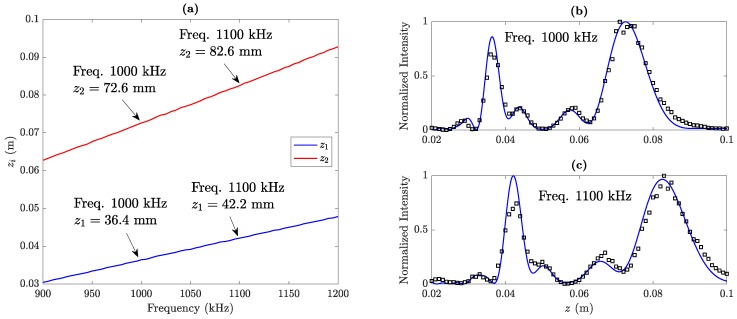
(**a**) Numerically computed focal distances as a function of the working frequency; Experimental (black squares) and simulated (blue line) focusing profiles at (**b**) 1 MHz and (**c**) 1.1 MHz.

Figure 9 shows the effect that the adjustment of the operating frequency has on the performance of the MbZP lens. Figure 9a depicts the location of both foci against the operating frequency for the built MbZP shown in Figure 7a. This MbZP lens has been designed to operate at a design frequency of f=1 MHz, and the results shown in Figure 9a have been numerically computed using Equation (6). As it can be observed from the figure, both foci shift linearly with the operating frequency, although at different rates. Thus, the separation between both foci augments with the operating frequency. Figure 9b,c shows the experimental MbZP focusing profiles measured for comparison purposes at f=1 MHz (design frequency) and f=1.1 MHz, respectively. The separations between both foci are around the numerically computed values, which correspond to 36.2 mm in the f=1 MHz case and 40.4 mm in the 1.1 MHz case. Therefore, Figure 9b,c experimentally demonstrates the effect that the operating frequency has on the MbZP focusing profile.

## 4. Conclusions

In this work, we present a method for designing bifocal ZPs using M-bonacci binary sequences in the ultrasound domain. The distance between both foci is related to the M-bonacci sequence ratio. In addition, a γ design parameter is presented, which can be used to finely adjust the foci separation at the expense of increasing the focusing profile distortion. Experimental results agree with numerical simulations, demonstrating the viability of these type of lenses in high-wavelength domains, such as microwaves or ultrasound transmission.

## Figures and Tables

**Table 1:** M-bonacci sequences for different *m* values.

*m*	$F_{m,j}$
2	{0,1,1,2,3,5,8,13,21,34}
3	{0,1,1,2,4,7,13,24,44,81}
4	{0,1,1,2,4,8,15,29,56,108}

**Table 2:** Examples of M-bonacci binary sequences.

*j*	m=2	m=3	m=4
1	{0}	{0}	{0}
2	{1}	{1}	{1}
3	{10}	{10}	{10}
4	{101}	{1010}	{1010}
5	{10110}	{1010101}	{10101010}
6	{10110101}	{1010101101010}	{101010101010101}
